# The Association of Anticoagulation Intensity with Outcomes in Hospitalized COVID-19 Patients

**DOI:** 10.1155/2024/8838308

**Published:** 2024-03-11

**Authors:** Rena Zheng, Alexandra Solomon, Madeline DiLorenzo, Iniya Rajendran, Joseph Park, Vrushali Dhongade, Michael A. Garcia, Robert T. Eberhardt, John Mark Sloan, Janice Weinberg, Elizabeth S. Klings

**Affiliations:** ^1^UMass Chan Medical School, UMass Medical Center, Department of Medicine, Division of Hematology-Oncology, Worcester, MA, USA; ^2^Eastern Vascular Associates, Denville, NJ, USA; ^3^New York University Grossman School of Medicine, Department of Medicine, Division of Infectious Diseases and Immunology, New York, NY, USA; ^4^University of Arizona, College of Medicine, Department of Medicine, Division of Cardiology, Tucson, AZ, USA; ^5^Brigham and Women's Hospital, Department of Medicine, Boston, MA, USA; ^6^Brigham and Women's Hospital, Department of Neurology, Boston, MA, USA; ^7^Valley Medical Center Pulmonary & Sleep Disorder Clinic, Covington, WA, USA; ^8^Boston University Chobanian & Avedisian School of Medicine, Boston Medical Center, Department of Medicine, Section of Cardiovascular Medicine, Boston, MA, USA; ^9^Boston University Chobanian & Avedisian School of Medicine, Boston Medical Center, Department of Medicine, Section of Hematology & Medical Oncology, Boston, MA, USA; ^10^Boston University School of Public Health, Department of Biostatistics, Boston, MA, USA; ^11^Boston University Chobanian & Avedisian School of Medicine, Boston Medical Center, Department of Medicine, The Pulmonary Center, Boston, MA, USA

## Abstract

Venous thromboembolism (VTE) risk is increased in patients infected with severe acute respiratory syndrome coronavirus 2 (SARS-CoV-2). A key question was whether increased intensity of anticoagulation would help prevent VTE and improve patient outcomes, including transfer to the intensive care unit (ICU) and mortality. At the start of the coronavirus disease-19 (COVID-19) pandemic, our institution, Boston Medical Center, instituted a VTE risk stratification protocol based on patients' initial D-dimer levels, medical history, and presence of thrombosis to determine whether they should receive standard-dose prophylaxis, high-dose prophylaxis, or therapeutic anticoagulation. We performed a retrospective observational cohort study examining the association of degree of anticoagulation with outcomes in 915 hospitalized COVID-19 patients hospitalized initially on the general inpatient wards between March 1,^,^ 2020, and June 1, 2020. Patients directly hospitalized in the ICU were excluded. Most, 813 patients (89%), in our cohort were on standard-dose prophylaxis; 32 patients (3.5%) received high-dose prophylaxis; 70 patients (7.7%), were treated with therapeutic anticoagulation. VTE occurred in 45 patients (4.9%), and the overall in-hospital mortality rate was 5.4% (49 deaths). On multivariable analysis of clinical outcomes in relation to type of anticoagulation, in the high-dose prophylaxis group, there was a trend towards increased in-hospital mortality (odds ratio 2.4 (0.8–7.5, 95% CI)) and increased ICU transfer (odds ratio 2.2 (0.9-5.7, 95% CI)). Our results suggest that patients receiving high-dose prophylaxis had more severe disease that was not mitigated by intermediate-dose anticoagulation.

## 1. Introduction

In hospitalized patients infected with coronavirus disease 2019 (COVID-19), venous thromboembolism (VTE) was common. A systematic meta-analysis of VTE events, including pulmonary embolism (PE) and deep vein thrombosis (DVT), during the first half of 2020 estimated the frequency of VTE to be 4–9% on inpatient wards and 7–24% in the intensive care unit (ICU) [1]. Published autopsy data from 12 patients who died early in the pandemic from COVID-19 demonstrated undiagnosed DVTs in 7 (58%) of these patients [2]. The true incidence of venous and arterial thromboses including VTE, ischemic stroke, and myocardial infarction (MI), varied widely in different cohorts. The rates of VTE and associated complications appear to be highest among patients requiring ICU care, ranging from 10 to 46%, even among those receiving thromboprophylaxis [3–5]. The pathophysiology for the observed increased frequency of VTE in COVID-19 patients is not completely understood. COVID-19 infection produces marked systemic inflammation, and many inflammatory and procoagulant-circulating proteins are elevated in patients who develop VTE, including D-dimer, fibrinogen, factor VIII, erythrocyte sedimentation rate (ESR), and C-reactive protein (CRP) [3, 5, 6].

In the spring of 2020, many institutions instituted changes in their VTE anticoagulation (AC) prophylaxis and treatment protocols for hospitalized patients to address the concern for increased VTE risk in COVID-19 patients. A retrospective study of over 4000 hospitalized COVID-19 patients demonstrated no significant difference between prophylactic and therapeutic AC on in-hospital mortality or the need for mechanical ventilation [7]. A smaller randomized phase II trial comparing therapeutic vs. prophylactic enoxaparin in patients requiring mechanical ventilation demonstrated that patients were more likely to be liberated from mechanical ventilation if treated with therapeutic enoxaparin [8]. The ACTIV-4 trial randomized more than 2000 noncritically ill and 1000 critically ill COVID-19 inpatients to therapeutic vs. prophylactic AC. There was a benefit of therapeutic anticoagulation only in noncritically ill patients, with increased organ support-free days [9, 10].

In April 2020, the Boston Medical Center (BMC) developed an empiric VTE risk stratification protocol for hospitalized COVID-19 patients utilizing serum D-dimer levels and presence of prior/current thrombosis. We hypothesized that a high-intensity prophylactic dose of anticoagulation would be protective against VTE in those deemed to have increased VTE risk. In this current retrospective observational cohort study, we sought to determine the impact of these guidelines on need for ICU care, organ failure, hemorrhagic complications, and in-hospital mortality.

## 2. Methods

### 2.1. Sources for Selecting Participants

A retrospective observational cohort study was performed using data from the Clinical Data Warehouse (CDW), the electronic medical record, and the Viral Infection and Respiratory Illness Universal Study (VIRUS) Registry of Patients in the ICU at BMC. Approval to conduct this study was granted by the BMC and the Boston University Medical Campus Institutional Review Board under protocol # H-40461.

### 2.2. Methods for Selecting Participants: Inclusion and Exclusion Criteria

Patients were identified for individual chart review using a search of the CDW database for adults (aged 18 years and older) with a positive nasopharyngeal polymerase chain reaction (PCR) test for SARS-CoV-2 hospitalized at BMC between March 1, and June 1, 2020. Only patients who did not initially require the ICU level of care upon hospitalization were included in this study. This allowed for ascertainment of the impact of the intervention on the need for ICU care.

### 2.3. Intervention Studied

**Figure 1:**
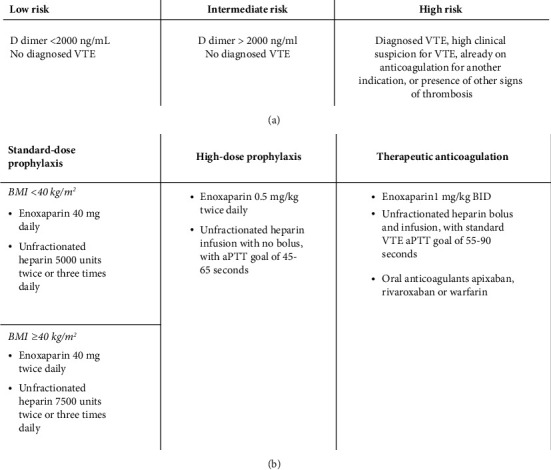
Stratification of risk of thrombosis in COVID-19 patients. (a) Definition of low-, intermediate- and high-risk patients infected with COVID-19. (b) Anticoagulation regimen used in standard-dose prophylaxis, high-dose prophylaxis, and therapeutic anticoagulation groups, with the number of patients per group.

On April 24, 2020, BMC launched a COVID-19 AC protocol once the prothrombotic effects of COVID-19 infection had begun to be recognized. This protocol consisted of guidelines that were recommended for all hospitalized COVID-19 patients and was enacted nearly halfway through the retrospective study period. All COVID-19 patients were risk stratified into low-, intermediate-(D-dimer >2000 ng/mL (8 times the upper limit of normal at our institution)), or high-risk (actual or suspected VTE or prior indication for therapeutic AC) groups (Figure 1(a)). Patients in the low-risk category were treated with standard-dose prophylactic AC which consisted of enoxaparin 40 mg daily or unfractionated heparin (UFH) 5000 units twice or three times daily for those with BMI <40 kg/m^2^. Standard-dose prophylaxis for those with BMI ≥40 kg/m^2^ consisted of enoxaparin 40 mg twice daily or UFH 7500 units twice or three times daily. Patients in the intermediate-risk category were treated with high-intensity prophylactic AC which consisted of either enoxaparin 0.5 mg/kg twice daily or intravenous UFH without a bolus and an activated partial thromboplastin time (aPTT) goal of 45–65 seconds. Patients in the high-risk group were treated with therapeutic dose enoxaparin (1 mg/kg twice daily) or UFH bolus and intravenous infusion, with an aPTT goal of 55–90 per standard clinical practice (Figure 1(b)). Patients with a prior history of therapeutic AC use upon admission were typically continued on their outpatient regimen. Data were obtained pre- and post-implementation of this protocol with comparisons of VTE frequency, need for ICU level care, organ failure, and in-hospital mortality. An assessment of hemorrhagic complications was performed, and their rate was compared among low-, intermediate-, and high-risk patients.

### 2.4. Outcomes, Exposures, and Predictors of Outcomes

Individual chart review was performed using standardized data collection forms. For each patient, demographics, prior medical history, medication usage, laboratory and radiologic data, and inpatient AC and COVID-19 specific treatment regimens were collected. VTE events were recorded and defined as follows: (1) DVT by Duplex ultrasound and/or (2) PE by computed tomography (CT) pulmonary angiogram. For patients with an ICU stay during their hospitalization, chart review was assisted using the VIRUS Registry which focused on those requiring ICU care. The specific outcomes evaluated were as follows: (1) need for ICU care, (2) use of mechanical ventilation, (3) episodes of hemorrhage, and (4) mortality.

### 2.5. Statistical Analysis

Descriptive statistics for categorical variables are reported as the number and percentage of total and for continuous variables as the median and 25^th^–75^th^ percentile (interquartile range). Data were analyzed using Fisher's exact test for categorical variables. Kruskal–Wallis tests were used to establish significance for continuous variables. Multivariable analysis examined the association between outcome and AC regimen, with odds ratios (OR) and 95% confidence intervals (CI) reported. Standard-dose prophylaxis was used as the control group. Potential confounders and effect modifiers were considered. Transfer to ICU was adjusted for age, current alcohol use, prior history of VTE, prior AC use, and hydroxychloroquine and azithromycin use reflective of COVID-19 treatment at the time of the study. To adjust for potential selection bias, in-hospital mortality was adjusted for age, current alcohol use, history of systemic hypertension, and hydroxychloroquine and remdesivir use. Vasopressors and mechanical ventilation were age-adjusted only, as the sample size was not large enough to adjust for other variables. All statistical analyses were performed using SAS version 9.4 (SAS Institute Inc., Cary, NC, USA). A two-tailed *p* value <0.05 was considered statistically significant. Variables with missing values are indicated in table footnotes. No variable with missing data was included in statistical models; therefore, no imputation was performed.

## 3. Results

### 3.1. Study Population

**Figure 2:**
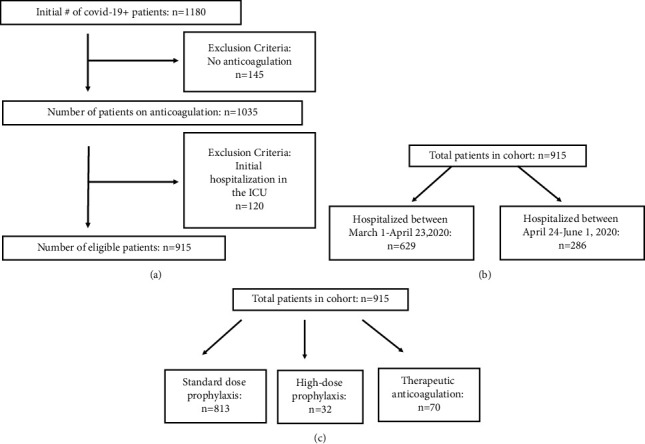
Study population. (a) The number of patients included and excluded to determine the final number of eligible patients. (b) The number of patients separated by time of hospitalization. (c) The number of patients in each anticoagulation-dose group.

A total of 1180 patients were eligible based on hospitalization with positive SARS-CoV-2 PCR. After excluding patients not on any anticoagulation, 1035 patients remained (Figure 2(a)). After excluding patients directly hospitalized in the ICU, 915 patients that received any type of AC and were initially hospitalized on the general wards at BMC between March 1, 2020, and June 1, 2020, were included in this study. Of these, 629 patients (68.7%) were hospitalized prior to the start of the COVID-19 AC protocol on April 24, 2020 (Figure 2(b)). Most patients (813, 88.9%) received standard VTE prophylaxis (Figure 1(b)), 32 (3.5%) received high-dose prophylaxis, and 70 (7.7%) were treated with therapeutic-dose AC (Table 1, Figure 2(c)). The median age of the entire cohort was 58 years (interquartile range 45–69 years). The patients in the high-dose prophylaxis and therapeutic-dose AC were older with a median age of 62 years (interquartile range 51–70 years) and 65.5 years (interquartile range 58–77 years), respectively, compared to 58 years (interquartile range 45–69 years, *p* < 0.0001) in the standard prophylaxis group. The cohort had 46.5% female and 46% Black, and the median BMI was 29.1 kg/m^2^ (interquartile range 24.9–34).

The patients in the therapeutic AC group were more likely to have a prior history of VTE (24.3%) than in the standard-dose (4.2%) and high-dose prophylaxis (12.5%) groups (*p* < 0.0001). A greater proportion of patients in the therapeutic AC group were treated with AC prior to hospitalization (60%) than those in the standard-dose (3%) and high-dose prophylaxis groups (12.5%) (*p* < 0.0001). There was no significant difference in prior antiplatelet agent use among groups. Those with current alcohol use were less likely to receive therapeutic AC (7%) than either standard- (20.9%) or high-dose prophylaxis (31.3%) (*p*=0.0225). Other medical comorbidities, including current tobacco use, diabetes mellitus, and hypertension, were similar across all three AC groups. There was a significant difference in the D-dimer level which was expected due to the design of the study (*p* < 0.0001), but there was no difference in CRP among the three AC groups.

Patients treated with therapeutic AC had higher rates of in-hospital VTE, as expected, since the majority of these patients were treated with therapeutic AC due to the presence of thrombosis detected on imaging. The overall rate of VTE in the entire cohort was 4.9%. The rate of VTE was 20% in the therapeutic AC group, compared with 3.6% in the standard-dose and 6.3% in the high-dose prophylaxis groups.

### 3.2. Treatment History

Patients received multiple treatments postulated to be beneficial in COVID-19 infection reflective of the time during which these data were collected. Two hundred eighty (30.6%) patients received a biologic agent including IL-6 receptor antagonists (tocilizumab or sarilumab), IL-1 antagonists (anakinra), or other monoclonal antibodies; there was no significant difference in the use of biologic treatments among the three AC groups (Table 2). Forty-three (4.7%) patients received dexamethasone, and 37 (4%) received remdesivir, similar across the three AC groups. In the initial phase of the pandemic, many patients were treated empirically with azithromycin and hydroxychloroquine. Four hundred thirty-eight (47.9%) patients received azithromycin, with no significant difference among the three AC groups. Five hundred fifty-six (60.8%) patients were treated with hydroxychloroquine; a higher percentage of patients received this treatment in the standard-dose prophylaxis group (62.9%, *p*=0.0003).

### 3.3. Clinical Outcomes

Forty-nine patients (5.4%) in the cohort died during their hospitalization (Table 3). There was a trend towards higher in-hospital mortality in the patients receiving high-dose prophylaxis (12.5%) or therapeutic AC (8.6%) than in the standard-dose prophylaxis group (4.8%, *p*=0.0594). One hundred twenty-seven patients (13.9%) in the entire cohort required ICU level care; this trended higher in those receiving high-dose prophylaxis (21.9%) and therapeutic AC (21.4%) than in those receiving standard-dose prophylaxis (12.9%, *p*=0.0555). Eighty-four (9.2%) patients required mechanical ventilation, and 75 (8.2%) required the use of vasopressors; no differences across treatment groups were observed.

**Figure 3:**
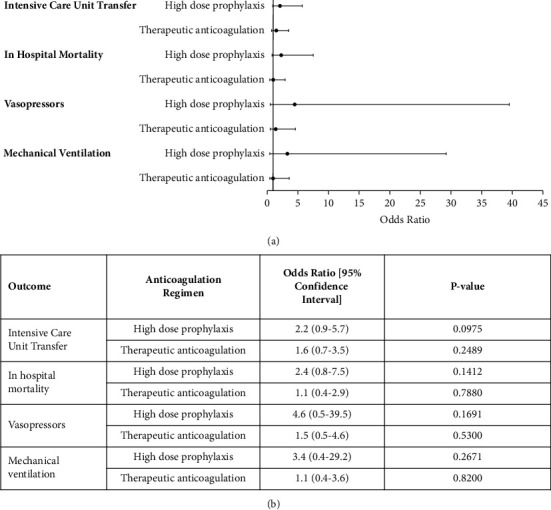
Multivariable analysis of clinical outcomes associated with the type of anticoagulation utilized. (a) Distribution of odds ratios with 95% confidence interval, showing outcomes for each type of anticoagulation. Standard-dose anticoagulation is the reference to which the other two regimens are compared. ^*∗*^*p*=0.0273 and ^*∗∗*^*p* < 0.0001. (b) Table with odds ratios with 95% confidence interval and *p* values corresponding to data points in the graph in figure (a). Significance assessed at *p* < 0.05.

Multivariable analysis demonstrated a trend towards higher intensive care utilization (OR 2.2, 95% CI 0.9–5.7), in-hospital mortality (OR 2.4, 95% CI 0.8–7.5), vasopressor (OR 4.6, 95% CI 0.5–39.5), and mechanical ventilation use (OR 3.4, 95% CI 0.4–29.2) in the high-dose prophylaxis group (Figures 3(a) and 3(b)).

### 3.4. Hemorrhagic Complications

The rate of significant hemorrhage in this cohort was low regardless of the AC regimen used (Table 3). Only 43 (4.7%) required packed red blood cell transfusion, and the rate of gastrointestinal (11, 1.2%) or intracranial hemorrhage (1, 0.1%) was low across the entire cohort.

## 4. Discussion

In our retrospective study of COVID-19 patients hospitalized on the general inpatient wards during the first three months of the pandemic, the frequency of VTE overall was 4.9% (45 patients), comparable to other retrospective studies [3, 11, 12]. Overall, the hemorrhagic risk was low. The mortality rate overall was 5.9% (49 patients), and the rate of ICU utilization was 13.9% (127 patients). Our mortality rate was lower than that observed in other studies, which has been reported to be as high as 24.5% in one study [11] ranging from 0.8% of general ward patients to 18.75% of critically ill patients in another study [3]. One possible reason for the lower observed mortality rates could be due to focusing on patients hospitalized on the general inpatient wards and not those requiring the ICU on presentation. Another possible explanation stems from the COVID-19 population at BMC. BMC is the largest safety net hospital in Boston, Massachusetts, and the age and racial background of our cohort is reflective of this. The median age of our cohort was less than 65 years. A meta-analysis of the mortality rate in COVID-19 patients has demonstrated that substantial increases in mortality from COVID-19 occur as people age, especially in those aged 60–69 or older [13]. It is possible that the younger age of our cohort conferred a survival advantage for our patients.

There was a trend towards higher odds of in-hospital mortality and ICU transfer in patients on both high-dose AC regimens, suggesting that these patients had greater degrees of inflammation leading to thrombosis risk or were more severely ill.

This patient cohort was part of the initial wave of COVID-19 infected patients when data regarding risk of thrombosis and impact of anticoagulation were not available. Elevation in D-dimer was associated with increased thrombosis risk and mortality [3]. Our data support this conclusion, as the patients in the high-dose prophylaxis group had the highest median D-dimer and a trend towards increased ICU transfer and in-hospital mortality.

There are limitations to this study. This study is limited by its retrospective design. The institution of the COVID-19 AC protocol guidelines was left to the discretion of individual physicians caring for these patients, and there was no mechanism in the current study design to distribute patients evenly among the risk groups. Baseline characteristics including age, current alcohol use, prior history of VTE, and AC use were not balanced across groups, although they were adjusted for as part of the multivariable analysis. Only a small percentage of our total patient population were already on outpatient AC (7.7%) prior to their admission, although in the therapeutic AC cohort, 60% were already on prior AC before admission. A retrospective study of 5392 hospitalized COVID-19 patients showed that those on warfarin had increased in-hospital mortality and those on prior direct oral anticoagulants had increased major bleeding incidence [14]. Our study showed that overall, there was a low bleeding incidence across all AC groups. Our study period covered the time both pre- and post-institution of our enhanced AC protocol, which may have contributed to the increased frequency of the cohort being treated with prophylactic AC. This impacted the sample size of the other two treatment groups which reduced the power to find significant changes in the results. We examined all DVTs and PEs during hospitalization, including on presentation, which limits our ability to determine whether there was a difference in VTE events after starting different doses of AC.

Our study population was a primarily underserved population in a major city hospital. The median age of the total cohort was 58 years and similar to the average age of patients in a randomized trial comparing therapeutic and prophylactic AC in noncritically ill patients [9]. This suggests our results may be generalizable to a nonelderly population. We have learned a lot about COVID-19 and hypercoagulability in the last three years, which continues to inform our understanding of thrombosis risk during COVID-19 infection. While we understand that COVID-19 infection may provoke thrombosis risk, there is not a clear prophylactic strategy. Our study needs to be taken into context of the period of time during which it was conducted (spring 2020) when there were little data regarding thrombosis prevention.

The largest randomized trial to date examining therapeutic compared with prophylactic AC in both critically ill and noncritically ill patients did show benefit in organ support-free days in the noncritically ill patients who received therapeutic AC [9]. There was no benefit observed in the critically ill patients. However, in this trial, there was not an intermediate anticoagulation dose, so it is unclear if this strategy would have had benefit. Our data support that intermediate-dose anticoagulation was not effective in mitigating poor outcomes in high-risk subgroups. Similarly, the INSPIRATION randomized clinical trial assigned 600 critically ill patients to either intermediate or prophylactic dose AC. There was no difference in outcomes based on a composite of thrombotic events, treatment with extracorporeal membrane oxygenation, and mortality [15].

As the pandemic has evolved, with emergence of different strains of SARS-CoV-2 and increased numbers of people who have been vaccinated against COVID-19, future studies should explore whether there has been any change in the risk of thrombotic complications. As the SARS-CoV-2 virus evolves, more definitive information regarding the role of AC intensity will be needed [16].

## Figures and Tables

**Table 1:** Subject characteristics.

Characteristic	Total (*n* = 915)	Standard-dose prophylaxis (*n* = 813)	High-dose prophylaxis (*n* = 32)	Therapeutic anticoagulation (*n* = 70)	*p* value
Age (yrs)	58 (45–70)	58 (45–69)	62 (51–70)	65.5 (58–77)	<0.0001
Female *n* (%)	416 (45.5)	378 (46.5)	11 (34.4)	27 (38.6)	0.1948
BMI (kg/m2)^†^	29.1 (24.9–34)	29.2 (24.9–34)	27.1 (24.9–30.9)	28.7 (23.3–35.2)	0.6809
Race					0.0072
Black, *n* (%)	421 (46)	364 (44.8)	14 (43.8)	43 (61.4)	
White, *n* (%)	153 (16.7)	130 (16)	8 (25)	15 (21.4)	
Other, *n* (%)	151 (16.5)	142 (17.5)	6 (18.8)	3 (4.3)	
Unknown, *n* (%)	190 (20.8)	177 (21.8)	4 (12.5)	9 (12.9)	
Comorbidities					
Current tobacco use, *n* (%)	117 (12.8)	105 (12.9)	5 (15.6)	7 (10)	0.6816
Current alcohol use, *n* (%)	187 (20.4)	170 (20.9)	10 (31.3)	7 (10)	0.0225
Diabetes mellitus, *n* (%)	323 (35.3)	285 (35.1)	12 (37.5)	26 (37.1)	0.8839
Hypertension, *n* (%)	547 (59.8)	478 (58.8)	18 (56.3)	51 (72.9)	0.0590
Prior history of venous thromboembolism, *n* (%)	55 (6)	34 (4.2)	4 (12.5)	17 (24.3)	<0.0001
Prior medication use					
Anticoagulants, *n* (%)	70 (7.7)	24 (3)	4 (12.5)	42 (60)	<0.0001
Antiplatelet agents, *n* (%)	216 (23.6)	188 (23.1)	7 (21.9)	21 (30)	0.4111
Inflammatory markers					
Initial D-dimer (ng/mL)^†^	317 (189–603)	310 (185–547)	2346.5 (601–3360.5)	357.5 (208–834)	<0.0001
Initial CRP (mg/L)^†^	65.6 (24.9–127.5)	65.6 (24.6–124.6)	84.9 (26.6–153.3)	64.9 (28.1–125.7)	0.3570
In-hospital thrombosis					
Venous thromboembolism, *n* (%)	45 (4.9)	29 (3.6)	2 (6.3)	14 (20)	<0.0001

Data are median (25th–75th percentile) for age, BMI, initial D-dimer, and initial CRP. All other variables are *n* (%) where *n* is the total number of patients. *p* values comparing all three anticoagulation groups are from Fisher's exact test or Kruskal–Wallis test. ^†^There are fewer *n* for the prophylactic group in that variable. For BMI, *n* = 811; for initial D-dimer, *n* = 786; for CRP, *n* = 797 for the prophylactic group.

**Table 2:** Medical treatments for COVID-19 infection.

Treatment	Total (*n* = 915)	Standard-dose prophylaxis (*n* = 813)	High-dose prophylaxis (*n* = 32)	Therapeutic anticoagulation (*n* = 70)	*p* value
Biologic, *n* (%)	280 (30.6)	243 (29.9)	15 (46.9)	22 (31.4)	0.1311
Dexamethasone, *n* (%)	43 (4.7)	36 (4.4)	3 (9.4)	4 (5.7)	0.2601
Remdesivir, *n* (%)	37 (4)	29 (3.6)	4 (12.5)	4 (5.7)	0.0328
Azithromycin, *n* (%)	438 (47.9)	398 (49)	13 (40.6)	27 (38.6)	0.1832
Hydroxychloroquine, *n* (%)	556 (60.8)	511 (62.9)	10 (31.3)	35 (50)	0.0003

Treatments patients underwent during hospitalization. Biologic is a conglomerate of IL-6 receptor antagonists (sarilumab or tocilizumab), IL-1 antagonist (anakinra), or any other monoclonal antibody that was used. Data are *n* (%) where *n* is the total number of patients. *p* values comparing all three anticoagulation groups are from Fisher's exact test.

**Table 3:** In-hospital outcomes.

Outcome	Total (*n* = 915)	Standard-dose prophylaxis (*n* = 813)	High-dose prophylaxis (*n* = 32)	Therapeutic anticoagulation (*n* = 70)	*p* value
In-hospital mortality, *n* (%)	49 (5.4)	39 (4.8)	4 (12.5)	6 (8.6)	0.0594
Intensive care unit transfer, *n* (%)	127 (13.9)	105 (12.9)	7 (21.9)	15 (21.4)	0.0555
Mechanical ventilation, *n* (%)	84 (9.2)	68 (8.4)	6 (18.8)	10 (14.3)	0.6431
Vasopressors, *n* (%)	75 (8.2)	59 (7.3)	6 (18.8)	10 (14.3)	0.3111
Need for packed red blood cell transfusion, *n* (%)	43 (4.7)	35 (4.3)	4 (12.5)	4 (5.7)	0.0833
Gastrointestinal hemorrhage, *n* (%)	11 (1.2)	8 (1.)	1 (3.1)	2 (2.9)	0.1372
Intracranial hemorrhage, *n* (%)	1 (0.1)	0 (0)	1 (3.1)	0 (0)	0.0350

Data are *n* (%) where *n* is the total number of patients. *p* values comparing all three anticoagulation groups are from Fisher's exact test.

## Data Availability

Original datasets are not made publicly available due to patient privacy.
